# Metastatic Renal Cell Carcinoma to the Scalp: A Case Report With Review of Literature

**DOI:** 10.7759/cureus.34790

**Published:** 2023-02-09

**Authors:** Huda A Meshikhes, Raihanah S Al Khatem, Hassan M Albusaleh, Ali A Alzahir

**Affiliations:** 1 Medicine and Surgery, Imam Abdulrahman Bin Faisal University, Dammam, SAU; 2 Surgery, King Fahad Specialist Hospital, Dammam, SAU

**Keywords:** surgical excision, scalp lesion, nephrectomy, skin metastases, renal cell carcinoma

## Abstract

Renal cell carcinoma (RCC) is the most common type of renal neoplasm. It accounts for 3% of solid tumors in adults and mostly affects men with the peak incidence between the fifth and seventh decades. It metastasizes mainly through the hematogenous spread, and the lung is the most common site of metastasis followed by bone, lymph node, liver, brain, and adrenal glands. Skin metastasis is extremely rare and accounts for <7% of RCC metastases, with the scalp and face being the most reported sites. Skin metastases are usually diagnosed at a later stage of the disease, commonly post-nephrectomy, and are regarded as a poor prognostic factor. Here we report a case of a 54-year-old male who presented with a red, pedunculated, bleeding, and nontender scalp lesion (2x2cm in size) found on the right parietal area. with a history of left radical nephrectomy and adjuvant chemotherapy for clear cell RCC 17 years ago, as well as laminectomy and radiotherapy for bone metastases in C5 and C6 in 2015. After surgical excision of the scalp lesion, histology revealed metastatic clear cell RCC. The patient was doing well post-surgical excision and was referred back to oncology where palliative care and supportive treatment were initiated. In the span of five months post-resection, he developed several conditions where his health further deteriorated. He was announced dead in September 2022 due to cardiac arrest. This case highlights the occurrence of scalp metastases long after the surgical resection of RCC.

## Introduction

Renal cell carcinoma (RCC) is the most common type of renal malignancy. It accounts for over 3% of all adult solid malignancies [1]. It has several histological subtypes, the clear cell being the most common [2] and is more prevalent among males with increasing incidence in the elderly population above the fifth to seventh decades [3,4].

RCC is often diagnosed incidentally during radiologic imaging and only about 10% of patients present with the classical triad of hematuria, flank pain, and a palpable mass [4]. RCC metastasizes primarily through hematogenous spread [5]. It is estimated that 33% of patients with RCC develop metastasis [6], and the lung is the most common site of metastasis followed by bone, lymph nodes, liver, brain, and adrenal glands [5]. Skin metastasis is extremely rare, and its presence is considered a poor prognostic factor [7].

We report here a case of RCC metastasizing to the scalp, 17 years after nephrectomy and adjuvant chemotherapy. Furthermore, we reviewed the literature on the clinical evaluation and management of similar cases.

## Case presentation

**Figure 1:**
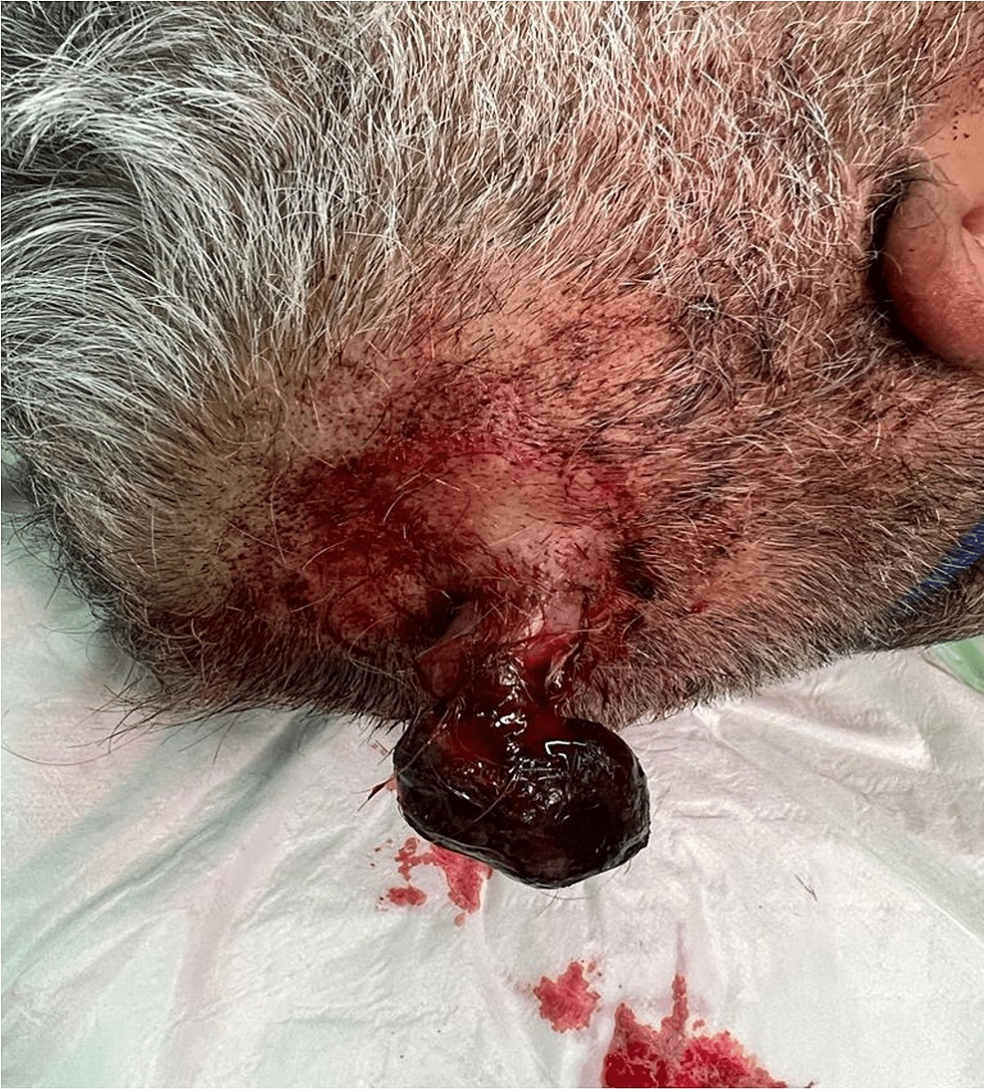
The scalp lesion before the surgical excision.

**Figure 2:**
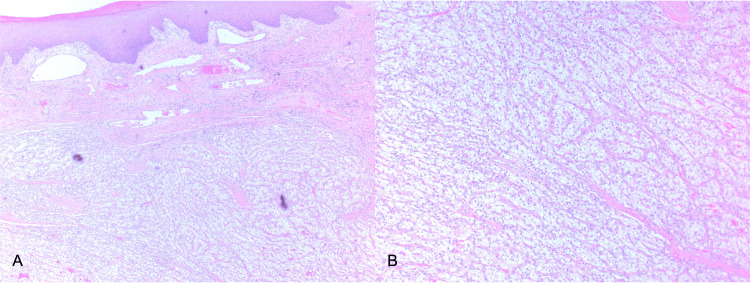
Histological micrograph showing skin with underlying renal cell carcinoma comprising cords for clear cells with a rich vascular background, magnification 4X (2A) and 20X (2B).

A 54-year-old male known case of type II diabetes mellitus, hypertension, dyslipidemia, and stage V chronic kidney disease on hemodialysis. In 2005, he underwent a left radical nephrectomy for incidentally found RCC, clear cell type, grade 2 out of 4, TNM stage pT3apN not assigned (no nodes submitted or found). Ten years later, he developed lytic bone metastases in C5 and C6 vertebrae. Laminectomy was performed and he was started on radiotherapy was completed in March 2015. Palliative Suntinib was started in July 2015 but then switched to Pazopanib in January 2017 at 400 mg once daily and escalated gradually to 800 mg once daily. Pazopanib was continued at a dose of 600 mg once daily but then stopped in March 2020 due to ischemic heart disease. He was referred by his oncologist to our clinic with a history of scalp swelling which was noticed two years earlier and was increasing in size over the past three months. On examination, a red pedunculated and pulsatile mass on the right parietal area of the scalp was noted. The mass was about 2x2cm in size, mobile, non-tender, and bled easily on scratching or touching (Figure 1). Complete surgical excision was performed under local anesthesia and the histology revealed metastatic clear cell RCC grade 2 with negative margins (Figures 2A, 2B). He was doing well after the removal of the scalp lesion and was referred back to the oncology team and decided to go for palliative care and supportive treatment.

The patient's health deteriorated five months after the excision, and he was admitted multiple times. He further developed bilateral peripheral neuropathy due to uncontrolled diabetes, right internal jugular vein thrombosis, and encephalopathy secondary to infection. CT brain was done and showed brainstem infarctions with no metastasis noted. Whole body PET/CT scan showed no local recurrence or distant metastases. During his last admission, he was transferred to the intensive care unit and was intubated as his Glasgow coma scale dropped to 6/15. Unfortunately, he passed away from cardiac arrest in September 2022.

## Discussion

Metastatic RCC to the skin is very rare and accounts for 1%-6.5% [1,2] of all RCC metastases with the scalp and face being the most prominent sites [3]. Our review of the literature revealed a total of 25 reported cases of RCC metastases to the scalp. The mean age of the patients was 57.4 (range: 30-83) years, and most of them were males (80%). Thirteen cases were post-nephrectomy for RCC before the presentation of their scalp lesions [1,4-14]. However, three patients have confirmed the diagnosis of RCC without nephrectomy [15-17]. The mean time interval between the treatment of the primary RCC and the diagnosis of the scalp lesion was 33 (range: 0-180) months. In nine patients, the first presentation was scalp metastasis [2,3,12,18-21], and five of the nine cases were subsequently diagnosed and confirmed with RCC after nephrectomy [2,18-21]. Table 1 summarizes the data of all reported cases of metastatic RCC to the scalp (from 1977 to 2022).

**Table 1:** Summary of reported cases of renal cell carcinoma metastatic to the scalp.

Pt No.	Author	Age (Years)	Sex	First presentation	Treatment to primary tumor	Location of skin metastasis	Location of other metastases	Treatment of skin metastasis	Time interval to skin metastasis (Months)	Survival after detection of skin metastasis (Months)
1	Abbasi, et al [1]	42	M	Flank pain, intermittent gross hematuria	Nephrectomy and chemotherapy	Scalp	None	Surgical excision	1	-
2	Yang, et al [4]	83	F	Incidental renal mass	Nephrectomy	Scalp	Lung, left iliac bone, spine	Surgical excision	180	-
3	Altinkaya, et al [15]	77	M	Flank pain and fatigue	Chemotherapy	Scalp	Lung, diaphragmatic pleura, neck lymph nodes, mediastinum, abdomen	Surgical excision	144	5
4	Krogerus, et al [18]	65	M	Scalp lesion	Nephrectomy	Scalp	Lung	Surgical excision	0	-
5	Anzalone, et al [16]	52	M	-	Chemotherapy	Scalp	-	Surgical excision	30	24
6	Leve, et al [6]	75	M	Incidental renal mass	Nephrectomy	Scalp	None	Surgical excision	84	-
7	Errami, et al [7]	64	M	Incidental renal mass	Nephrectomy	Scalp	Tonsils, cervical lymphadenopathy, bone	Chemotherapy	36	-
8	Snow, et al [8]	69	F	Acute onset hematuria	Nephrectomy	Scalp	Bicep, mediastinum, spine	Surgical excision	72	-
9	Smyth, et al [9]	67	M	-	Nephrectomy	Scalp	Left ureter, rectus abdominus muscle, pancreas, brain	Surgical excision	120	9
10	Ferhatoglu, et al [10]	40	F	-	Nephrectomy	Scalp	None	Surgical excision	14	-
11	Selvi, et al [11]	51	M	-	Nephrectomy	Scalp, distal phalanx	Oral cavity, brain, lung	Surgical excision	36	6
12	Kishore, et al [17]	54	M	-	Palliative therapy	Scalp, upper lip	Bone, retromolar, mediastinum, bilateral hilar lymphadenopathy, lung, left adrenal	Chemotherapy, radiotherapy	2	-
13	Dorairajan, et al [12]	55	M	-	Nephrectomy	Scalp, cheek	Abdomen, brain	Interferon	10	4
14		55	M	Scalp lesion	None	Scalp	Liver, bone	None	0	3
15		30	M	Scalp lesion	None	Scalp	Liver, bones, brain	Surgical excision	0	3
16		40	M	Scalp lesion	None	Scalp	Lung	Interferon	0	8
17		56	M	-	Nephrectomy	Scalp	Bones	None	19	7
18	Estrada-Chavez, et al [5]	80	M	-	Nephrectomy	Scalp	None	None	48	-
19	de Paula, et al [3]	61	M	Incidental renal mass	Nephrectomy was not done due to heart failure decompensation	Scalp, dorsum, face	Lung	Surgical excision	0	Dead from acute respiratory failure
20	Pan, et al [2]	63	M	Scalp lesion	Nephrectomy	Scalp	None	Surgical excision	0	-
21	Eke, et al [19]	42	F	Scalp lesion, hematuria	Nephrectomy	Scalp	None	None	0	Lost to follow-up
22	Singla, et al [13]	53	M	Weight loss, flank mass	Nephrectomy, chemotherapy	Scalp	Upper jaw	Surgical excision, chemotherapy	17	-
23	Gandla, et al [14]	45	M	-	Nephrectomy	Scalp	Adrenal	Radiotherapy	12	-
24	Wahner-Roedler, et al [21]	72	F	Scalp lesion	Nephrectomy	Scalp	None	Radiotherapy	0	15
25	Livingston, et al [20]	45	M	Scalp lesion	Nephrectomy	Scalp	None	Surgical excision	0	-
26	Our case	54	M	Incidental renal mass	Nephrectomy	Scalp	Spine	Surgical excision	204	Cardiac arrest

The clinical presentation of the scalp metastasis was either only a red to purple, firm, and well-defined mass or accompanied by other characteristics, such as itching, bleeding, painless, erythematous, vegetated, ulcerated, or expanding pulsatile lesion. Because cutaneous metastasis of RCC mimics other common dermatological conditions such as hemangioma, cutaneous angiosarcoma, basal cell carcinoma, melanoma, or pyogenic granuloma, it is challenging to diagnose it at an early stage [22]. This requires physicians to have a high index of suspicion for such cases.

Despite substantial research into the methods of dissemination of RCC, the precise process is still unknown. Hematogenous metastasis, lymphogenous dissemination through the thoracic duct, direct invasion, or implantation from procedures are a few suggested pathways. The major mechanism of metastasis is thought to be through the hematogenous spread. The highly vascular nature of these tumors allows the cells to spread to several organs via the renal vein. For instance, the pathway for metastasis to the lung is from the renal vein to the vena cava reaching the right atrium to the lungs. It has been postulated that the valveless vertebral veins (plexus of Batson) act as a conduit for RCC to travel from the renal vein to the emissary scalp veins ultimately reaching the scalp and skin [4,15,23,24].

Partial or radical nephrectomy is the mainstay management of RCC, and it is considered a cytoreductive strategy in patients with metastatic RCC [25]. For most cases in the literature, surgical excision was done for the scalp mass [1-4,6,8-13,15,16,18,20]. Our patient was similarly managed by excising the scalp lesion. However, in some instances where surgical excision is not an option, chemotherapy, radiotherapy, or interferon are considerable alternatives [7,12,14,17,20,21].

Since metastatic RCC is becoming refractory to conventional chemotherapy, more advanced targeted therapy has emerged including sorafenib, sunitinib, bevacizumab, pazopanib, and lenvatinib. These agents are regarded as tyrosine kinase inhibitors that function by inhibiting the vascular endothelial growth factor (VEGF) signaling axis. Sunitinib and pazopanib are approved as first-line management, while the mTOR inhibitors, everolimus, and temsirolimus, are accepted as second-line treatment [25]. Another option is the use of interleukin-2 and interferon alfa, however, these cytokines showed lower response rates and shorter progression-free survival compared to sunitinib (5%-20% vs. 30%-40%) [26]. Our patient was started on sunitinib after undergoing laminectomy of C5 and C6 but switched to pazopanib due to poor compliance. It was found in a study by Motzer et al that pazopanib, despite having similar efficacy to sunitinib, performed better in terms of safety and quality of life [27]. 

The presence of skin metastasis from RCC is considered a poor prognostic factor where up to 90% are associated with synchronous visceral metastases [28]. The median survival of metastatic clear cell RCC is about 13 months with five-year survival under 10% [29,30]. When the literature was reviewed, 10 patients died within a mean of 8.4 months after the detection of skin metastasis [9,11,12,15,16,21].

## Conclusions

In conclusion, metastatic RCC to the scalp needs a high index of suspicion to detect. Patients are mostly diagnosed with scalp metastasis in a later stage of their disease even after nephrectomy. The management plan for these cases is usually surgical excision, but when it is not feasible, chemotherapy, radiotherapy, or interferon are alternative options. Although skin metastasis of RCC is a rare occurrence, it may warrant the incorporation of a follow-up strategy as it carries a poor prognosis.
